# Surgical treatment of Dupuytren’s disease – outcome and health economy in relation to smoking and diabetes

**DOI:** 10.1186/1471-2474-15-117

**Published:** 2014-04-02

**Authors:** David Eckerdal, Axel Nivestam, Lars B Dahlin

**Affiliations:** 1 Department of Clinical Sciences Malmö - Hand Surgery, Lund University and Skåne University Hospital, Malmö, Sweden

**Keywords:** Dupuytren’s contracture, Diabetes, Smoking, Fasciectomy, Surgery

## Abstract

**Background:**

The conventional treatment for Dupuytren’s disease is surgery. The introduction of alternative treatment strategies creates a need to track outcomes and costs relating to surgical treatment and risk factors, such as smoking and diabetes. This was the aim of the present study.

**Methods:**

In a prospective study, the outcome of open surgical treatment for finger flexion contracture in Dupuytren’s disease (175 patients; 182 surgical procedures) was studied by evaluating valid QuickDASH forms answered by subjects before surgery and one year postoperatively. Data were also obtained from medical records, and preoperative declarations concerning health.

**Results:**

In all subjects (median [25% - 75% percentiles] age 68 [62-73]), the QuickDASH score improved from 22 [9-36] to 5 [0-18]. Smokers (27/179 procedures) were younger and had a more severe degree of disease and dysfunction preoperatively than non-smokers, but the outcome of surgery did not differ between the groups. Subjects with diabetes (20/181 procedures) were younger than those without diabetes, but their disease severity or outcome did not differ. Hand specialists operated faster than residents, but the surgical outcome did not differ. Healthcare costs for surgery for Dupuytren’s contracture were $ 2392 (€ 1859), which were not higher among smokers or subjects with diabetes. Only 22 patients remained in hospital (2 [1-2.3] days) and 28 patients needed sick leave (28 [21-31] days). The occurrence of necrosis of skin flaps (12%) or infections (6%) was no more frequent among smokers or those with diabetes.

**Conclusions:**

There is no difference in surgical outcome for finger flexion contracture in Dupuytren’s disease between smokers and non-smokers or between subjects with or without diabetes, although smokers had more severe preoperative contracture. The costs for surgical treatment for finger flexion contracture in Dupuytren’s disease should be viewed in relation to that for other treatment strategies.

## Background

Dupuytren’s disease is a condition that affects various parts of the palmar aponeurosis in the hands, but it can also occur in the sole of the foot and in the penis. The disease results in a progressive formation of nodules and cords in the palm leading to a flexion contracture, usually affecting the metacarpophalangeal (MCP-) and proximal interphalangeal (PIP-) joints in the fingers involved [1]. Flexion contracture may have a great impact on daily living due to the functional difficulties, e.g. difficulty in washing the face or putting on gloves [2].

Dupuytren’s disease has a genetic component [3] and can manifest with varying severity. It commonly affects men, however, women also often present, but with a different disease character, such as isolated contracture of the pip-joint in the little finger, and may also have a later disease onset [4]. There is a higher prevalence of Dupuytren’s disease in a population of subjects with diabetes, while impaired glucose tolerance (IGT) does not seem to increase the prevalence of the disease [5], although this has been questioned [1]. Subjects with diabetes, however, may have a higher incidence of postoperative complications [6] and the outcome of surgery in such patients has been barely studied. Smoking has also been a contested factor in Dupuytren’s disease, with some studies showing a correlation between the occurrence of Dupuytren’s disease and smoking [7], but again the outcome of surgery in smokers has not been particularly emphasized.

Treatment of Dupuytren’s disease to date has been open surgery (e.g. partial fasciectomy, fasciotomy, dermofasciectomy or amputation) and in some parts of Europe, e.g. France and the Nordic countries, the more unusual percutaneous needle fasciotomy is performed [8,9]. Over recent years a new pharmacologic treatment, injection of a collagenase into the fibrous cords with a subsequent “pop up” procedure to break the Dupuytren’s disease cord, has become available and is now common, despite being more expensive than percutaneous needle fasciotomy [9,10]. A recurrence of Dupuytren’s disease after treatment is common, and is a result of the method of treatment [11,12]. The costs and resources required for various surgical treatments have not so far been evaluated.

Our purpose was to evaluate the outcome, assessed using the QuickDASH questionnaire [13], of surgical treatment of Dupuytren’s disease as well as the severity of the preoperative flexion contracture in relation to the risk factors of smoking and diabetes. In addition, the healthcare costs for various components of the treatment, such as pre- and post-operative outpatient visits, open surgery, rehabilitation and medical treatment by nurses, were evaluated. Such costs are important in view of the recent introduction on the market of a pharmacological substance to treat finger flexion contracture in subjects with Dupuytren’s disease [9].

## Methods

In this prospective study, subjects treated for finger flexion contracture due to Dupuytren’s disease, at the Department of Hand Surgery, Skåne University Hospital, Malmö, Sweden between September 2009 and February 2011, were identified by searching the administrative records for the diagnosis code Dupuytren’s contracture (ICD 10 code M72.0).

Subjects treated surgically during this time period were routinely provided with the QuickDASH form to be filled in before and one year after the surgical procedure. A Swedish translation of the form, including eleven questions measuring daily life symptoms and function of the arm, was used. Each question was judged by the patient from 1 (no disability) to 5 (great disability). The total score was calculated and varied between zero and 100. At least ten questions had to be answered for the form to be valid [13,14].

In addition, healthcare costs for various treatments, such as outpatient or surgery visits, rehabilitation, and treatment by nurses, were estimated. Lengths of hospital stay and sick leave were calculated among the subjects who were admitted to the hospital and those still in the labour market, respectively.

### Subjects

To be included, subjects had to be surgically treated for finger flexion contracture due to Dupuytren’s disease between September 2009 and February 2011 and to have completed a valid QuickDASH form both before and one year after surgery. Subjects who were operated on primarily for another main disorder of the hand were excluded. Subjects were divided according to whether they underwent an open surgical procedure or a percutaneous procedure. Only subjects who had an open surgical procedure were included (n = 182). Subjects with procedures, such as arthrodesis or amputations, were excluded.

Preoperative information was registered from the subjects’ declaration on the health form, which is routinely collected before surgery, while other pre- and post-operative data were obtained from the medical records of the subjects. Additional data, such as age, gender, body mass index (BMI), disease severity (i.e. finger flexion contracture), duration of surgical procedure, presence of postoperative infections and or necrosis of skin flaps (including delayed healing), were also collected. Three subjects were excluded due to insufficient information obtainable from the records. When available, the preoperative QuickDASH, as well as the above information, were also collected from the records of those subjects who had been excluded because of the lack of or an invalid postoperative QuickDASH. Thus, allowed comparison between excluded and included patients.

The severity of the finger flexion contracture due to Dupuytren’s disease was graded based on the extension deficit of the subject. The following revised model of the Tubiana staging system was used:

Stage 0: no lesion, healthy

Stage N: palmar or digital nodule without established flexion deformity

Stage Ia: total flexion deformity between 0° and 20°

Stage Ib: total flexion deformity between 21° and 45°

Stage II: total flexion deformity between 45° and 90°

Stage III: total flexion deformity between 91° and 135°

Stage IV: total flexion deformity exceeding 135° (7).

### Calculation of costs for treatment

The costs for treatment were calculated using the following figures (Prices from Skåne University Hospital; 2012; exchange rate for US dollar ($) and Euro (€) calculated from mean value from 2012): $20 (€ 16) for each minute of surgery, $ 187 (€ 144) for each appointment with the surgeon, $ 75 (€ 58) for each visit to the rehabilitation unit or to the nurses at the outpatient clinic and $ 513 (€ 397) for each day spent on the ward.

### Statistical methods

Values are presented as median [IQR; 25 – 75 percentiles]. Nominal data are shown as numbers (%). The chi-square and the Mann-Whitney tests were used to detect any significant differences between groups. P-values < 0.05 were considered statistically significant. IBM SPSS Statistics, version 20 for Mac and StatView for Windows (SAS institute Inc., version 5.0.1, Cary NC, USA) were used for all calculations.

### Ethical considerations

The present project, and those with similar design, is not covered by the law (2003:460) in Sweden - “The ethical review of research involving humans” – according to the Ethical Review Board in Lund and no advertising is needed. The study has been done in compliance with the Helsinki Declaration (http://www.wma.net/en/30publications/10policies/b3/index.html).

## Results

During the period covered by the study 268 patients at the department received any treatment primarily for Dupuytren’s disease in the form of any procedure to correct finger flexion contracture due to the disease. In total 288 different procedures were performed. In three of these patients/procedures no data were found or recorded in the subjects’ medical records. Thus, three subjects were not available for analyses, resulting in a selected group of 265 patients and 285 procedures.

A valid QuickDASH form was completed by 195 subjects (74%) and was available for 202 of the procedures (71%). Seven of the 20 bilaterally treated subjects handed in a valid QuickDASH form for both hands. Thus, the group of subjects that could tentatively be studied comprised 202 procedures (i.e. 171 fasciectomies, eleven fasciotomies, ten percutaneous needle or knife fasciotomies and six amputations/arthrodesis; 4 unknown procedure).

### Included subjects with preoperative and postoperative valid QuickDASH

Most of the subjects had an open surgical procedure; i.e. 182/202 (90%) of the selected procedures were open surgery (i.e. open fasciectomies or open fasciotomies); 16 (8%) procedures were either a percutaneous needle or knife fasciotomy or an amputation/arthrodesis; in the remaining four (2%) procedures the method of treatment was unknown. Patients with amputation/arthrodesis were too few to be included in the open surgery group and were also interpreted as having a procedure different from partial fasciectomy and open fasciotomy; thus were excluded from the open procedures and the number of procedures included were n = 182.

### Characteristics of all included subjects and results of open surgery

The characteristics of the included subjects/surgeries are presented in Table 1. Among the 182 open procedures (i.e. open fasciectomies and fasciotomies), most were performed in men (median age 68 years). In 80 (44%) of these procedures, the subject had a BMI > 25, classified by WHO as overweight [15]. Twenty of the procedures were performed in subjects with type 1 (10%) or type 2 (85%) diabetes (5% had an unknown type of diabetes; in one patient information about glucose status was lacking). Eighty-three (46%) procedures were performed in subjects who were receiving pharmacological treatment for hypertension and the subjects in 36 (20%) procedures were being treated with statins. In 27 (15%) of the procedures, the subjects reported that they were smokers (i.e. information about smoking was lacking in three patients).

**Table 1:** Subjects’ characteristics in 182 open surgical procedures for finger flexion contracture in Dupuytren’s disease

	**All surgeries (n = 182)**	**Non-smokers**^**a **^**(n = 152)**	**Smokers**^**a **^**(n = 27)**	**p-value**	**Subjects without diabetes**^**b **^**(n = 161)**	**Subjects with diabetes**^**b **^**(n = 20)**	**p-value**
Age	68 [62-73]	70 [64-75]	63 [59-67]	**0.001**	69 [63-74]	63 [59-70]	**0.03**
Gender	Male (78%)	Male (75%)	Male (93%)	**0.04**	Male (79%)	Male (80%)	0.83
BMI	25.1 [23.4-27.1]	25.1 [23.4-27.1]	25.3 [23.1-26.8]	0.87	24.9 [23.1-26.8]	26.8 [23.7-28.9]	0.06
Hypertension (treated) (%)	46%	46%	41%	0.71	42%	75%	**0.001**
Statin treatment (%)	20%	20%	19%	0.88	17%	40%	**0.0001**
Preoperative flexion contracture (degrees)	90 [60-110]	85 [60-105]	98 [79-143]	**0.04**	90 [60-110]	80 [63-113]	0.95
Postoperative flexion contracture (degrees)	20 [0-35]	20 [0-30]	30 [11-40]	0.12	20 [0-35]	20 [15-35]	0.57
Improvement (degrees)	65 [45-87]	60 [45-85]	78 [40-91]	0.60	65 [45-90]	60 [45-80]	0.55
Preoperative Tubiana stage	1b [20 (11%)]	1b [19 (12%)]	1b [1 (4%)]	0.09	1b [19 (12%)]	1b [1 (5%)]	0.97
	2 [81 (45%)]	2 [69 (45%)]	2 [10 (37%)]		2 [70 (43%)]	2 [10 (50%)]	
	3 [41 (22%)]	3 [36 (24%)]	3 [5 (18%)]		3 [37 (23%)]	3 [4 (20%)]	
	4 [18 (10%)]	4 [12 (8%)]	4 [6 (22%)]		4 [16 (10%)]	4 [2 (10%)]	
	Unknown [22 (12%)]	Unknown [16 (11%]	Unknown [5 (19%)]		Unknown [19 (12%)]	Unknown [3 (15%)]	
Postoperative Tubiana stage	0 [34 (19%)]	0 [29 (19%)]	0 [4 (15%)]	0.66	0 [31 (19%)]	0 [3 (15%)]	0.98
	1a [37 (20%)]	1a [33 (22%)]	1a [4 (15%)]		1a [32 (19%)]	1a [5 (25%)]	
	1b [49 (27%)]	1b [38 (25%)]	1b [11 (41%)]		1b [43 (27%)]	1b [6 (30%)]	
	2 [10 (5.5%)]	2 [9 (6%)]	2 [1 (4%)]		2 [9 (6%)]	2 [1 (5%)]	
	3 [1 (0.5%)]	3 [1 (1%)]	3 [0 (0%)]		3 [1 (1%)]	3 [0 (0%)]	
	4 [0 (0%)]	4 [0 (0)]	4 [0 (0%)]		4 [0 (0%)]	4 [0 (0%)]	
	Unknown [51 (28%)]	Unknown [42 (28%)]	Unknown [7 (26%)]		Unknown [45 (28%)]	Unknown [5 (25%)]	

Values are median [25% - 75% percentiles], numbers (%) or %. ^a^Information about smoking was lacking in three patients. ^b^Information about glucose status was lacking in one patient.

In the group that underwent open surgery (n = 182), both pre- and post-operative Tubiana staging was available for 121 (66%) procedures. Preoperative Tubiana staging was present in 160 (88%) subjects, while 131 (72%) had postoperative Tubiana staging; thus, 14 (8%) of the subjects had no measurements. In most of the procedures, the subjects had the preoperative Tubiana stage “2” [n = 81 (45%)]. The Tubiana stage improved after surgery to 1b [n = 49 (27%)]; although postoperatively only 6% of the measured fingers had a Tubiana stage of “2” or higher. The median improvement in Tubiana score was 3 [2-4] stages. The extension deficit decreased considerably (Table 1). In 21 (12%) of the procedures, skin necrosis or delayed healing after surgery was observed to some extent. In 11 (6%) of the procedures, the subject was treated for a superficial infection in the wound.

### Outcome after open surgery among all included (n = 182) subjects

The scores of the QuickDASH pre- and post-operative (n = 182), as well as the change (including those with a postoperative score of >10 and a change < 10), are shown in Table 2. QuickDASH scores decreased after surgery among subjects in the whole population. In most of the 182 open procedures included, subjects had a postoperative change > 10 in QuickDASH score and most had a remaining QuickDASH scores < 10. Both these criteria are considered to be crucial in evaluating outcome, where change of more than > 10 is considered the minimal significant change and a postoperative score > 10 indicates remaining disability [16,17].

**Table 2:** QuickDASH scores in 182 open surgical procedures for finger flexion contracture in Dupuytren’s disease

	**All surgeries (n = 182)**	**Non-smokers**^**a**^**(n = 152)**	**Smokers**^**a **^**(n = 27)**	**p-value**	**Subjects without diabetes**^**b **^**(n = 161)**	**Subjects with diabetes**^**b **^**(n = 20)**	**p-value**
**Preoperative QuickDASH (total score)**	22 [9-36]	21 [9-34]	30 [14-43]	**0.04**	21 [9-36]	30 [7-43]	0.25
**Postoperative QuickDASH (total score)**	5 [0-18]	5 [0-16]	5 [0-25]	0.90	5 [0-18]	7 [2-17]	0.48
**Change in QuickDASH (total score)**	11 [2-24]	10 [2-22]	11 [5-25]	0.29	11 [2-23]	10 [3-30]	0.58
**Postoperative QuickDASH > 10 (numbers; %)**	65 (36%)	53 (35%)	11 (41%)	0.56	57 (35%)	7 (35%)	0.40
**Change in QuickDASH total score < 10 after surgery (numbers; %)**	74 (41%)	65 (43%)	8 (30%)	0.20	64 (40%)	9 (45%)	0.43

Values are median [25% - 75% percentiles] or numbers (%). ^a^Information about smoking was lacking in three patients. ^b^Information about glucose status was lacking in one patient.

### Excluded subjects (n = 83) due to incomplete preoperative or postoperative QuickDASH

In 83/285 procedures (i.e. no data in patient folder in three procedures), the subjects did not hand in two valid QuickDASH forms and were thus excluded from the study. Of these, 34 (41%) had a valid preoperative QuickDASH. There were no significant differences between the data from the excluded and the included subjects. The data of the excluded subjects are *not* included in other parts of the Results (below) or in any of the Tables.

### Surgically treated smokers and non-smokers

The characteristics of the subjects and the outcomes after open surgical procedure in smokers and non-smokers are presented in Tables 1 and 2. Smokers were younger than non-smokers and the preoperative extension deficit in the worst affected finger was more severe in smokers than in non-smokers. However, the preoperative extension deficit, as evaluated by Tubiana scoring, did not differ between the two groups.

As shown in Table 2, smokers had higher preoperative QuickDASH scores compared to non-smokers. Postoperatively, there were no significant differences in any outcome variables between smokers and non-smokers. However, there was a significant difference in the length of stay on the ward, with the smokers having a shorter stay than the non-smokers (Table 3). There was no difference between smokers and non-smokers in postoperative occurrence of skin necrosis or infections [16 (11%) and 5 (19%); p = 0.23, respectively] or infections [9 (6%) and 2 (7%); p = 0.77, respectively].

**Table 3:** Costs for treatment in 182 open surgical procedures for finger flexion contracture in Dupuytren’s disease

	**All surgeries (n = 182)**	**Non-smokers**^**a **^**(n = 152)**	**Smokers**^**a **^**(n = 27)**	**p-value**	**Subjects without diabetes**^**b **^**(n = 161)**	**Subjects with diabetes**^**b **^**(n = 20)**	**p-value**
**Duration of operation (min)**	77 [56-102]	74 [56-103]	86 [55-108]	0.22	79 [57-102]	68 [52-101]	0.28
**Appointments with the surgeon (numbers)**	3 [2-4]	3 [2-4]	3 [2-3]	1.00	3 [2-4]	2 [2-3]	0.18
**Appointments with hand therapist (numbers)**	2.5 [0-5]	2 [0-5]	3 [0-5.5]	0.32	2 [0-5]	3 [0-4.3]	0.95
**Appointments with nurse (numbers)**	1 [0-1]	1 [0-1]	1 [0.3-1]	0.33	1 [0-1]	1 [0.3-1]	0.27
**Sick leave (days)**^**1**^	28 [21-31]	28 [21-31]	28 [21-37]	0.94	28 [21-31]	30 [28-34]	0.27
**Duration of hospital stay (days)**^**2**^	2 [1-2.3]	2 [2-3]	1 [1-1]	**0.01**	2 [2-3]	1 [1-1.8]	0.11

Values are median [25% - 75% percentiles]. ^a^Information about smoking was lacking in three patients. ^b^Information about glucose status was lacking in one patient.

^1^Only from those procedures when subjects were sick listed.

^2^Only for those procedures when the subject was admitted to a ward.

### Surgically treated subjects with and without diabetes

The characteristics of the subjects and the outcomes of open surgery among those with and without diabetes are presented in Tables 1 and 2. The subjects with diabetes were younger than the patients without diabetes, while BMI did not differ significantly. As expected, the presence of treated hypertension differed significantly between patients with and without diabetes being more common in subjects with diabetes, and a similar pattern was also observed for treatment with statins. There were no differences between subjects with or without diabetes in the postoperative occurrence of skin necrosis [19 (12%) and 2 (10%); p = 0.81, respectively] or infections [10 (6%) and 1 (5%); p = 0.83, respectively].

There were no significant differences between subjects with and without diabetes in pre- or post-operative severity of flexion contracture or in improvement after surgery expressed in degrees. Furthermore, the Tubiana stages did not differ between the two groups. In addition, as shown in Table 2, QuickDASH scores did not differ between the groups. Finally, no other healthcare variables differed (Table 3).

### Costs and resources in surgically treated subjects

A specialist in hand surgery performed 65% [n = 118] of the procedures, while a resident did fewer [n = 62 (34%)]. The duration of surgery performed by specialists was 10 minutes shorter, but there were no differences in any variables or outcomes between subjects operated on by a specialist or a resident.

Data for resources, without costs, of included patients (n = 182) are shown in Table 3 and costs are presented here. The median duration of the surgical procedure in all the patients resulted in an individual median cost of $ 1572 (€ 1222). Appointments with the surgeon incurred a median cost of $ 557 (€ 433) in each subject. Visits to the rehabilitation unit resulted in a median cost of $ 188 (€ 146), while treatments by a nurse resulted in a median cost of $ 75 (€ 58). Thus, the median total health care cost was $ 2392 (€ 1859) for each subject in the total population. Most subjects were day-case procedures [n = 151 (83%)]. Those who were admitted [n = 22 (12%); 2 (1-2.3) nights] incurred in a median additional cost of $ 1022 (€ 795) per subject. Thus, the total healthcare cost for admitted subjects was $ 3414 (€ 2654). Most procedures were performed on subjects who were retired [n = 123 (68%)]. The subjects who needed sick leave [n = 28 (15%)] spent 28 [21-30.8] days off work, which resulted in an income loss of $ 4303 (€ 3345) per subject on sick leave.

## Discussion

The present data clearly indicate that open surgery has a favourable effect for finger flexion contracture in Dupuytren’s disease at one-year post surgery. In addition, the data concerning characteristics of the subjects are consistent with a previous study with a larger population of subjects [18], indicating that our population is relevant in European context. However, one should keep in mind that recurrences are common after surgical treatment of finger flexion contracture in Dupuytren’s disease [12]; an aspect that is not addressed or was an aim in the present study. The present study also reveals that smokers, who were younger than non-smokers, were treated at a more severe stage of the disease, but smoking as a risk factor did not have any negative impact on the outcome of the surgical treatment of finger flexion contracture in Dupuytren’s disease. Furthermore, subjects with diabetes were also younger than those without diabetes, but again the outcomes did not differ. Specialists in hand surgery operated ten minutes faster than residents, but outcomes after open surgery were no different, irrespective of the presence of the risk factors smoking or diabetes among the subjects.

### Smoking and surgical treatment of finger flexion contracture in Dupuytren’s disease

In our study, 15% of the patients were smokers, which correlates well with a larger European study in which the number of smokers with Dupuytren’s disease in the Nordic region was 18% [18]. In the rest of Europe, percentages of smokers with Dupuytren’s disease differ widely, i.e. 41-49% smokers [18]. Interestingly, the data in that European survey also indicate that the stage of Dupuytren’s disease in the treated subjects was more severe in the Nordic countries, which is consistent with a tradition that the indication for surgery in the Nordic region is a more advanced Tubiana stage or even that smoking speeds up the progression of the disease.

The effects of smoking on Dupuytren’s disease are contested [7,19]. The present data indicate that smokers are treated at a later stage of Dupuytren’s disease, but we have no explanation for this observation. It may be caused by factors related to the subjects, i.e. individual factors or a direct pathophysiological mechanism(s), or to the surgeons, or both. The smokers had a higher preoperative QuickDASH score, although they were younger than the non-smokers. One may speculate that Dupuytren’s disease develops earlier in smokers than in non-smokers; thus, smoking may be a risk factor for development of finger flexion contracture in Dupuytren’s disease. In one study, where a connection between Dupuytren’s disease and smoking was shown, the risk was adjusted for age [7]; we did not make any adjustments of that sort in our study. Nevertheless, there were no significant differences in postoperative QuickDASH score or in improvement of QuickDASH score between smokers and non-smokers, indicating that open surgical treatment can be performed later without jeopardizing the outcome, the postoperative Tubiana score or the extension deficit. In addition, the present smokers did not have a higher percentage of complications, such as necrosis of skin flaps (including delayed healing) or infections, but one cannot entirely exclude the possibility that the limited number of subjects in the present study may have precluded the detection of any difference in complications. Therefore, the present findings should be interpreted with some caution and the results should be confirmed in larger populations from national registries.

### Diabetes and surgical treatment of finger flexion contracture in Dupuytren’s disease

Dupuytren’s disease is more frequently seen in subjects with type 2 diabetes [5], but the disease may be less severe and progress more slowly in subjects with diabetes than in healthy subjects [1]. Our treated subjects with diabetes were younger indicating, in accordance with other studies [1], that the disease occurs earlier in subjects with diabetes. The percentage of the present subjects with diabetes, consisting mainly of subjects with type 2 diabetes, was 11%, which was lower than in the European survey (17% in Nordic countries and 26-36% in the rest of Europe [18]), and implies that fewer subjects with diabetes are treated surgically at our department. However, there were no differences in any outcome variables between the subjects with and without diabetes, except that subjects with diabetes were younger. Again, this may indicate that Dupuytren’s disease develops earlier in patients with diabetes, but a favourable outcome is still to be expected. Subjects with diabetes may have a higher incidence of postoperative complications [1], but this is in contrast to our study results. Taken together, our results imply that despite an earlier development of the disease, diabetes does not impact the outcome of surgical treatment of finger flexion contracture in Dupuytren’s disease. This is notable in view of an estimated global increase in the number of subjects with diabetes and thus an expected worldwide increase in the need for treatment of Dupuytren’s disease in subjects with diabetes.

### Resources and costs for surgical treatment of finger flexion contracture in Dupuytren’s disease

The costs, if the subject was admitted, were higher [$ 2392 (€ 1859) compared to $ 3414 (€ 2654)] than if the procedure was performed as outpatient surgery. The length of stay at the ward in subjects who were admitted differed from that for northern Europe in general (i.e. one night), while it is longer in other parts of Europe [18]. A reduction in the number of unnecessary days on the ward may reduce costs for the healthcare system since admission to a ward in connection with a surgical treatment is expensive and resource intensive, which is an important point in view of a lower financial support to hospitals and an increased population globally. However, admission to a ward may be required if the procedure is a reoperation with a risk of surgical complications, or the subject has a complicated health condition (e.g. non-optimal value of PT/INR). Interestingly, smokers had a shorter stay at the ward and thus potentially lower costs. Finally, some of the treated subjects were still on the labour market and needed sick leave after the surgery, resulting in a loss of production; thus, a further cost for the society. The loss of production should be taken into account when considering the treatment of choice for a patient with finger flexion contracture due to Dupuytren’s disease in view of the shorter rehabilitation after percutaneous needle fasciotomy or pharmacological treatment (i.e. collagenase injections) of the contracture, but with the risk of a faster recurrence after the latter two procedures than after open surgical treatment. One should also consider the costs for collagenase treatment; i.e. costs for one vial of drug $ 951 (€ 739) in Sweden. It has been estimated that the average patient needs to treat 1.2 fingers with collagenase (personal communication Pfizer Inc., Sweden), which will result in a total costs for treatment of $ 1768 (€ 1374) [i.e. 1.2 fingers × (two outpatient visits + two visits to rehabilitation unit + cost collagenase vial) = total costs for collagenase treatment]. In accordance, the costs for a percutaneous needle fasciotomy would be $ 522 (€ 405) [i.e. one extended (*x*2) outpatient visit and two visits to rehabilitation unit]. However, the complexity of the disease, with various severity of the disease, makes it difficult to judge the exact costs for treatment in a simple way [20], since the recurrence after collagenase treatment and percutaneous needle fasciotomy may be faster than after surgery, which should be taken into account. Surgery may also require admission to a ward in some patients, where the other two procedures can be performed as outpatient procedures.

The surgical procedures that were performed by a specialist took 10 minutes less than those done by residents, but the pre- or post-operative clinical status did not differ, indicating that the severity of the Dupuytren’s disease was similar in the patients treated. In addition, the outcomes did not differ with respect to QuickDASH scores or improvement. These results are interesting from a cost point of view as well as logistically. One may suggest that more surgical procedures on cases of Dupuytren’s disease could be performed by residents, freeing more time for specialists to deal with more severe cases, but without jeopardizing outcomes.

### Limitations of the study

The data concerning the number of treatment sessions by nurses, physiotherapists and occupational therapists may *possibly* be an underestimation, since the data recorded in the medical charts may be incomplete and such categories of staff may have treated some subjects outside the department. A similar limitation, related to an underestimation of the costs, is that we had no information about how many outpatient visits the subjects made to their GP before referral, but approximately half of the subjects were referred from a GP. Around 10% of the subjects also wrote “their own referral letter” because of their symptoms and limitations on their activities due to finger flexion contracture caused by Dupuytren’s disease. The rest of the subjects were or had been treated for another hand condition at the department. Therefore, we could not with 100% certainty calculate the costs that included pre-referral costs. However, these are probably low in comparison with the treatment costs; i.e. total costs for treatment consisting of a) health care costs (e.g. outpatient visits to surgeon, nurses and hand therapists) and b) loss of production (e.g. sick-leave).

Another limitation is the time period (i.e. one-year follow up) during which the subjects were collected. Had the time period for data collection been longer a more accurate evaluation of costs could have been made. However, forms with declarations of health together with QuickDASH data were only available during the selected time period. Thus, in future it would be interesting to evolve the study to include a longer time period with more subjects, including a 100% cover of e.g. Tubiana staging, and additional relevant information about other treatment options, such as percutaneous needle fasciotomy or pharmacological treatment (i.e. collagenase injections). Such a focus, including long-term recurrence of the disease and contracture, could be achieved by assessing national registries to include a large number of subjects nationally (http://www.hakir.se).

The final limitation is that we used the generic assessment scale QuickDASH for evaluation of the treatment. Finger flexion contracture in Dupuytren’s disease has a distinct impact on the patients’ activities and quality of life [2] and at present there is no suitable assessment scale that is specific for this disease. We, therefore, used the QuickDASH questionnaire to evaluate the outcome of surgery. However, in the near future a specific scale for evaluating the outcomes of various treatment strategies will be developed [2].

## Conclusions

Surgical treatment is favourable in subjects with Dupuytren’s finger flexion contracture. The outcomes of surgical treatment of Dupuytren’s contracture, including the frequency of complications, such as skin necrosis and infections, do not differ between smokers and non-smokers or between subjects with or without diabetes, although the preoperative finger flexion contracture was more severe in the former. Furthermore, smokers and subjects with diabetes were younger, which may indicate a tendency to develop Dupuytren’s disease earlier. The costs for surgical treatment of finger flexion contracture in Dupuytren’s disease should be seen in perspective in relation to the price of other treatment strategies.

## Abbreviations

MCP joint: Metacarpophalangeal joint; PIP joint: Proximal interphalangeal joint; IQR: Interquartile range (25^th^ – 75^th^ percentiles); ICD: International statistical classification of diseases and related health problems; WHO: World Health Organization; BMI: Body mass index; SEK: Swedish kronor; PT/INR: Prothrombin time/international normalized ratio.

## Competing interests

DE and AN have no conflicts of interest. LD has been a member of the advisory board for Pfizer Inc. and Auxilium Inc. and has been a PI in three clinical trials of Xiapex® in Sweden.

## Authors’ contributions

Concept/design LD; Data collection AN, DE; Data analysis/interpretation AN, DE, LD, Drafting, critical revision and approval of article AN, DE, LD; Statistics AN, DE, LD. All authors read and approved the final manuscript.

## Pre-publication history

The pre-publication history for this paper can be accessed here:

http://www.biomedcentral.com/1471-2474/15/117/prepub
